# New insights into the function of Interleukin-25 in disease pathogenesis

**DOI:** 10.1186/s40364-023-00474-9

**Published:** 2023-04-01

**Authors:** Qingfang Yuan, Na Peng, Fan Xiao, Xiaofei Shi, Bo Zhu, Ke Rui, Jie Tian, Liwei Lu

**Affiliations:** 1 Institute of Medical Immunology, Affiliated Hospital of Jiangsu University, Zhenjiang, China; 2 Department of Immunology, Jiangsu Key Laboratory of Laboratory Medicine, School of Medicine, Jiangsu University, Zhenjiang, China; 3 Department of Rheumatology, the Second People’s Hospital, Three Gorges University, Yichang, China; 4 Department of Pathology, Shenzhen Institute of Research and Innovation, The University of Hong Kong, Chongqing International Institute for Immunology, Chongqing, China; 5 Centre for Oncology and Immunology, Hong Kong Science Park, Hong Kong, China; 6 Department of Rheumatology and Immunology, The First Affiliated Hospital, School of Medicine, Henan University of Science and Technology, Luoyang, China; 7 Department of Laboratory Medicine, Affiliated Hospital of Jiangsu University, Zhenjiang, China

**Keywords:** IL-25(IL-17E), Autoimmune diseases, Inflammation, Cancer, Clinical treatment

## Abstract

Interleukin-25 (IL-25), also known as IL-17E, is a cytokine belonging to the IL-17 family. IL-25 is abundantly expressed by Th2 cells and various kinds of epithelial cells. IL-25 is an alarm signal generated upon cell injury or tissue damage to activate immune cells through the interaction with IL-17RA and IL-17RB receptors. The binding of IL-25 to IL-17RA/IL-17RB complex not only initiates and maintains type 2 immunity but also regulates other immune cells (e.g., macrophages and mast cells) via various signaling pathways. It has been well-documented that IL-25 is critically involved in the development of allergic disorders (e.g., asthma). However, the roles of IL-25 in the pathogenesis of other diseases and the underlying mechanisms are still unclear. This review presents current evidence on the roles of IL-25 in cancers, allergic disorders, and autoimmune diseases. Moreover, we discuss the unanswered key questions underlying IL-25-mediated disease pathology, which will provide new insights into the targeted therapy of this cytokine in clinical treatment.

## Introduction

IL-17 family cytokines have been shown to play a pivotal role in host defense against extracellular pathogens and inflammatory responses. Most members of the IL-17 family are disulfide-linked homodimers, with a molecular weight of the monomer ranging from 17 to 21 kDa. IL-17 family members can bind to its receptors for signaling, and each IL-17R subunit constitutes a unique protein [1–3]. IL-25, also named IL-17E, is a member of the IL-17 family and was originally discovered via a sequence alignment of human genomic DNA sequence data [1]. In humans, the *IL25* gene is located on the q-arm of chromosome 14 (14q11.2). Murine *Il25* is located on chromosome 7 and can encode 169 amino acids, with an 80% sequence homology to humans [1, 4]. Although IL-25 was initially found to be secreted primarily by T helper 2 (Th2) cells, a variety of other tissues and cells including lung and colon epithelial cells, alveolar macrophages, eosinophils, basophils, and mast cells have been identified as cellular source of IL-25 [1, 3, 4]. In addition, IL-25 functions to promote the expression of cytokines associated with type 2 immunity, such as IL-4, IL-5, IL-13, and thymic stromal lymphopoietin (TSLP) [1, 4–7]. The cellular targets of IL-25 include T cells, myeloid lineage cells, and non-hematopoietic cell populations (e.g., fibroblasts and mesenchymal cells) [1, 3, 4]. Previous studies have suggested a strong relationship between IL-25 and cancer, inflammation, and autoimmune diseases [8, 9]. Therefore, this review aims to provide a comprehensive summary of recent findings on the role of IL-25, encompassing topics such as:

- IL-25 receptors, related signaling pathways, cellular sources, and related cytokines.
- Roles of IL-25 in cancer, allergic conditions, and autoimmune diseases.
- IL-25 as a prospective target for clinical therapy.

## Introduction of IL -25

### IL-25 receptors

The IL-17 receptor (IL-17R) family consists of five receptor subunits, comprising IL-17RA-IL-17RE. The IL-25 receptors consist of a heterodimer composed of IL-17RA/IL-17RB [1–3]. IL-17RB (IL-17BR), also known as IL-17 receptor homolog 1 (IL-17Rh1) or EVI27, was the first identified receptor for IL-25. In previous studies, researchers found some new receptors that might be related to IL-17R. To identify candidate receptors, expressed sequence tags were examined for sequences related to IL-17R. On the basis of one such group of expressed sequence tags, a cDNA was found that encoded a 502-amino acid single transmembrane protein that shared 26% amino acid identity to IL-17R. The protein is then named IL-17Rh1 (IL-17RB) [1–3]. IL-17RB is expressed in kidney, liver and other peripheral organs. RNA and protein assays have revealed that there are two isoforms of IL-17RB, the membrane-bound type and the soluble type [3]. Subsequent research has shown that the IL-25 receptor also includes IL-17RA. Moreover, IL-17RA is the largest protein in the IL-17R family and possesses a unique TILL structural domain. Thus, IL-17RA is capable of binding to at least four ligands (IL-17 A, IL-17 C, IL-17E, and IL-17 F) for signaling. The presence of IL-17RA has been detected in mouse spleen, kidney, liver, lung, brain, heart, skeletal muscle, and testicular tissues, as well as in a wide variety of cell lines [3, 5]. Splenocytes from IL-17RA-knockout (KO) (*Il17ra*^*−/−*^) or IL-17RB-KO (*Il17rb*^*−/−*^) mice ceased to produce relevant inflammatory factors (e.g., IL-5 or IL-13) upon in vitro stimulation with IL-25. Following an intranasal injection of IL-25 in both types of the knockout mice, the inflammatory cells such as eosinophils, neutrophils, lymphocytes and macrophages did not show any changes. In addition, the lung tissue did not display any pathological alterations, including inflammatory cell infiltration [3, 6]. These results suggest that IL-25 signaling requires both IL-17RA and IL-17RB for downstream activation.

Each IL-17R subunit is a single transmembrane domain protein with multiple conserved patterns, such as extracellular fibronectin III-like motifs, transmembrane sections, and similar expression to fibroblast growth factor genes (SEF) /IL-17 receptor (SEFIR) domains in cytoplasmic [2, 3, 5]. After binding to IL-25, the heterodimer composed of IL-17RA/IL17-RB recruits SEFIR-containing proteins and further activates downstream signaling pathways involved in innate and adaptive immunity [2, 5, 6].

### IL-25 signaling pathway

Upon binding to IL-25, the IL-17RA/IL17-RB receptor complex can attract SEFIR-containing proteins, such as Act1 (NF-κB Activator 1, Nuclear Factor κB Activator 1), also known as CIKS. Act1 both exhibits E3 ubiquitin ligase activity and also includes a SEFIR structural domain containing a CC’ loop peptide [7, 10, 11]. When the SEFIR structural area is deleted in either of the Act1 or IL-17RB fragments, the interaction between Act1 and IL-17RB disappeared [12–14]. Moreover, previous studies have shown a distinctive reduction in the Th2 response and lung inflammation in Act1-deficient (*Traf3ip2*^−/−^) mice compared to the wild-type mice in a mouse model of allergic lung inflammation [12]. Further studies have shown that an Act1 deletion in epithelial cells could alleviate IL-25-induced allergic lung inflammation [12, 13, 15].

**Fig. 1:**
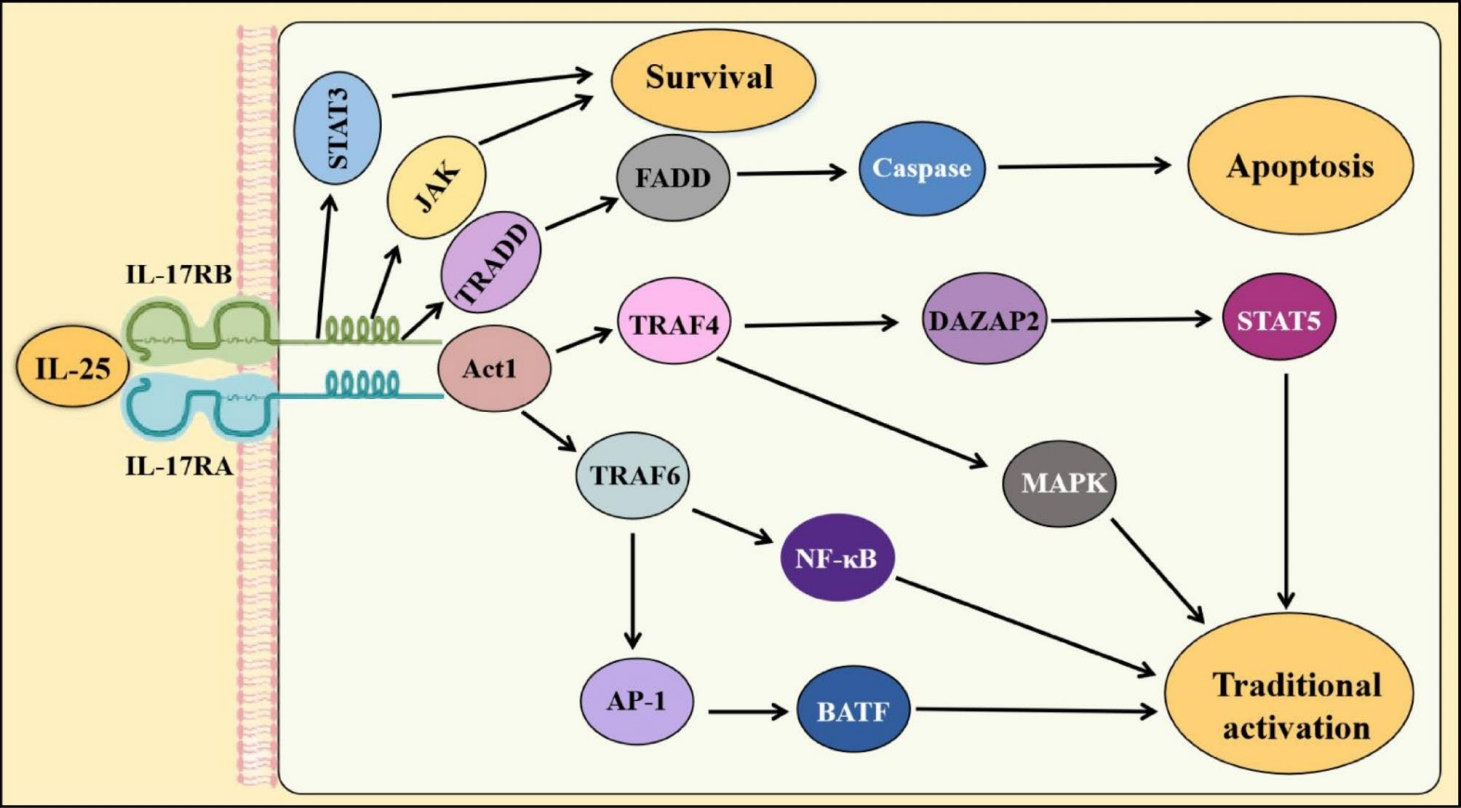
The signal pathway of IL-25. IL-25 binding to the receptor complex IL-17RA/IL-17RB can recruit adapter proteins, then mediate the signal transduction, such as Act1. Act1 can influence TRAF6 to induce inflammatory responses via the transcription factors NF-κB and AP-1. TRAF4 is also activated by Act1, which would activate not only the MAPK pathway but also its dependent DAZAP2 degradation leading to STAT5 recruitment to the IL-17RB subunit. TRADD and FADD are also recruited by the IL-25/IL-25R complex, and they mediate apoptosis. IL-25 might activate the JAK and STAT3 pathways, which are associated with cell survival. The image is produced by Adobe Illustrator

The tumor necrosis factor receptor-associated factor (TRAF) family members are also engaged in IL-25 signal transduction through Act1. Act1 recognizes and ubiquitinates TRAF adapters, which are subsequently recruited into the receptor complex to participate in the activation of downstream signaling pathways. In addition, Act1 recruits a substantial amount of TRAF6, which is essential for IL-25-mediated activation of the downstream nuclear factor-κB (NF-κB) pathway [16–18]. The binding of Act1 to TRAF6 also promotes the activation of the activator protein 1 (AP-1) downstream protein, basic leucine zipper transcription factor, activating transcription factor-like (BATF). In intestinal helminth infection, tissue-resident group 2 innate lymphoid cells (ILC2) modulated mucosal barrier homeostasis by responding to tuft cell–derived IL-25. BATF promoted IL-4 and IL-13 expression by ILC2 upon IL-25 stimulation, causing a type 2 immune response to alleviate inflammation. The aforementioned two pathways are essential for the participation of IL-25 in the progression of various diseases [19–21]. In contrast, IL-25 failed to activate the NF-κB pathway in *Traf6*^*−/−*^ mice [18] (Fig. 1).

TRAF4 is also activated by Act1 and participates in the activation of IL-25 signaling. Studies have shown that the expression of Th2 cytokines in the airways of *Traf4*^*−/−*^ mice is reduced, even with the stimulation of IL-25 [22, 23]. Moreover, TRAF4 can recruit E3 ligase smadubiquitin regulatory factor 2 to degrade Deleted in Azoospermia-associated protein 2 (DAZAP2), an IL-25R inhibitory factor. In contrast, silencing DAZAP2 increases the interaction between Act1 and IL-25R, as well as the responsiveness of IL-25. Therefore, TRAF4-Smadubiquitin regulatory factor 2 (SMURF2) -mediated DAZAP2 degradation represents a key initiating event for the IL-25 response. TRAF4-SMURF2-mediated DAZAP2 degradation following IL-25 stimulation can promote JAK2-mediated phosphorylation of Y444 and Y454, resulting in STAT5 recruitment to the IL-17RB subunit [22–24]. However, IL-25 failed to phosphorylate ERK1/2 and P38 in primary T cells and epithelial cells from *Traf4*^*−/−*^mice, and did not activate the MAPK pathway [22, 23] (Fig. 1).

IL-25 signaling can also be mediated by other pathways. For instance, the deletion of STAT5 was found to result in a defective Th2 response associated with IL-25 in epithelial cells [19, 25]. IL-25 has been reported to promote the proliferation of keratin-forming cells in the skin via the JAK1/2 and STAT3 pathways in a mouse model of psoriasis, inducing large amounts of inflammatory cytokines and chemokines in the skin of the mice [26, 27]. In investigations related to liver cancer, IL-25 was found to maintain the self-renewal of human cancer stem cells via the activation of STAT3 and NF-κB [26–29]. Moreover, IL-25 selectively recruits the tumor necrosis factor receptor 1 (TNFR1) associated death domain protein (TRADD) adapter protein and Fas-associated death domain (FADD), which consequently activate caspases and induce the apoptosis of target cells [2] (Fig. 1).

In conclusion, IL-25 can bind to the receptor and activate multiple downstream signaling pathways, including NF-κB, MAPK, JAK and STAT3, which play diverse roles in self-renewal, survival and apoptosis of cells as well as inflammation, tumor progression.

### Cellular sources of IL-25

**Fig. 2:**
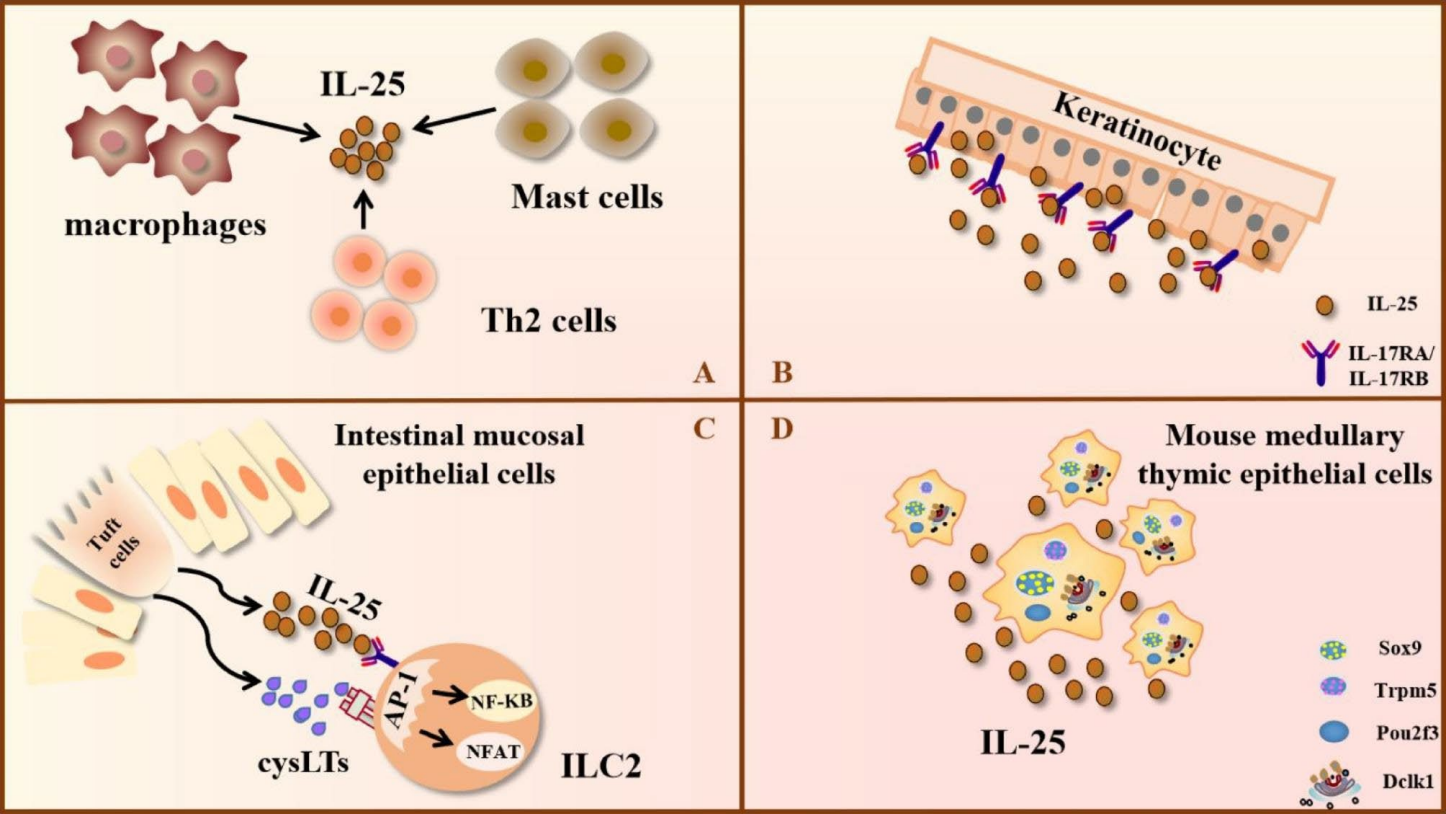
IL-25 is produced by various cell populations under different conditions. Th2 cells, mast cells, and macrophages are all capable of secreting IL-25 (Part A). Epithelial cells have been a major source of IL-25. Keratin-forming cells could produce IL-25 and IL-25R, which in combination further promote the progression of psoriasis (Part B). IL-25 and cysLTs from intestinal tuft cells could rapidly activate ILC2 by activating AP-1, followed by selective mobilization of NF-κB and NFAT, to clear the intestinal inflammation caused by helminth infection (Part C). One subpopulation of mouse thymic epithelial cells could generate IL-25 and further participate in thymic immunity (Part D). The image is produced by Adobe Illustrator

IL-25 was initially considered as a Th2 cell-derived factor, since the identification of its mRNA expression in highly differentiated Th2 cells in gastrointestinal tract and uterus of mice in 2000 [6]. Subsequently, the production of IL-25 by bone marrow-derived mast cells upon IgE crosslinking was discovered, thereby demonstrating that mast cells also produce IL-25 [30]. In addition, alveolar macrophages during particle induced lung inflammation produced IL-25. to promote lung inflammation [31]. Notably, activated eosinophils and basophils from allergic subjects could expressed IL-25. The IL-25 has biological activity and enhances the function of Th2 memory cells [7]. Further studies revealed that epithelial cells at different sites of inflammation could also express IL-25. The transcripts of IL-25 is present in skin keratinocytes [32, 33]. Subsequently, study found that keratin-forming cells from skin in psoriasis patients could express IL-25 and also express IL-25R [33] (Fig. 2).

Tuft cells are a type of intestinal epithelial cell that can secrete IL-25 [34–37]. Additionally, Tuft cells from the intestine can secrete cysteinyl leukotrienes (cysLTs) [38, 39]. Further studies have shown that IL-25 and cysLTs can selectively mobilize NF-kB and nuclear factor of activated T cells (NFAT) transcription factors for ILC2 activation [37–39] (Fig. 2). The above signaling cascade is essential for intestinal remodeling and helminth clearance. Interestingly, a subpopulation in mouse medullary thymic epithelium can express several characteristic makers including Dclk1, Sox9, Trpm5, and Pou2f3, and might secrete IL-25 in high quantities [35, 40, 41] (Fig. 2). Further investigation may provide in-depth understanding of the role of thymic tuft cells and IL-25 in shaping type 2 immune responses.

### Cytokines associated with IL-25 functionality

#### IL-17 A

IL-17 A has been the most frequently studied member of IL-17 cytokine family. IL-17 A is known to participate in the pathogenesis of multiple diseases (e.g., psoriasis, rheumatoid arthritis [RA], and contact hypersensitivity) [42–44]. Among the IL-17 family members, IL-25 appears to be the most diverged member of the family. IL-25 shares just 16% sequence homology with IL-17 A, while IL-17 F has the highest homology (55%) with it. IL-17B, IL-17 C, and IL-17D share sequence homology from 23 to 29% with IL-17 A [3, 45–47] (Table 1).

**Table 1:** Commonality and differences between IL-25 and IL-17 A

Comparative targets		IL-25	IL-17A	References
**Biological Features**	Homology	The homology of IL-25 to IL-17A is 16%.		[3, 45–47]
	Form of generation	In the form of disulfide-linked dimers.		[1–3]
**Cellular sources**		Th2 cells, mast cells, macrophages, etc.	Th17 cells	[1, 2]
**Factors associated with signalling pathways**	IL-17RA	The co-receptor		[5, 7]
	TRAF6	Participate in the activation of the NF-κB pathway.		[12, 18]
	Act1	Be recruited into IL-25 and IL-17A related signalling pathways.		[7, 10–14]
	TRAF4	Mediate signaling downstream.	Compete with TRAF6 for the TRAF binding domain on Act1	[22, 23]
**Performance in different diseases**	Psoriasis	Participate in promoting psoriasis progression.		[26, 50–54]
	Contact hypersensitivity	IL-25 promotes higher IL-17A expression		[44]
	Experimental autoimmune encephalomyelitis	IL-25 could work as a receptor antagonist of IL-17A function.		[59]
	Rheumatoid arthritis			[60]

Both IL-17 A and IL-25 can bind to IL-17RA and subsequently recruit Act1 to further activate the NF-κB pathway through the TRAF family [7, 12, 14–17, 26] (Table 1). However, the specific participating members of the TRAF family differ, whereas the same members are differentially expressed in the pathways downstream of IL-25 and IL-17 A. For example, as shown above, both TRAF4 and TRAF6 could participate and promote the activation of IL-25 downstream signaling pathways [12–18]. In addition to TRAF6, the downstream activation of IL-17 A combined with Act1 involves other members of TRAF family, including TRAF2, TRAF3, and TRAF5 [18, 22, 48, 49] (Table 1). However, TRAF4 functions as a negative regulator during IL-17 A-mediated signaling and inflammatory responses. The underlying mechanism has been shown to be due to TRAF4 competing with TRAF6 for the binding domain of TRAF on Act1 during IL-17 A downstream signaling. Therefore, the activation efficiency of both TRAF6 and the subsequent NF-κB pathway is substantially diminished [23] (Table 1).

Antimicrobial peptides produced by keratinocytes is the first line of host defense against infection. Previous studies have revealed that IL-17 A could assist in the host defense against pathogens (e.g., *Staphylococcus aureus*) in the skin. In vivo studies, IL-25 was different from IL-17 A. The host resistance to pathogens was not impaired in the mice deficient for IL-25 in the epidermis compared with the wild-type mice [50–54]. These results suggest that IL-25 dose not participate in the antimicrobial response and it also dose not affect the antimicrobial function of IL-17 A (Table 1).

In some specific cases, IL-25 can affect the expression of IL-17 A. Previous studies have demonstrated that while IL-17 A could induce and promote chronic skin inflammation, the precise mechanism remained unclear [45]. Further studies revealed that mice injected with recombinant mouse IL-25 were able to induce skin inflammation. Furthermore, *Il17a*^*−/−*^mice injected with recombinant mouse IL-25 still induced psoriasis. In the *Il25*^*−/*−^mouse model of psoriasis, the expression of IL-17 A was reduced, which may be attributed to the reduced recruitment of IL-17 A-producing γδ T cells in psoriasis-like skin inflammation. The above findings further demonstrated that IL-25 could induce psoriasis independently and participate in regulating IL-17 A in the progression of psoriasis [26, 50–54]. In contact dermatitis, IL-1β produced from skin dendritic cells could promote the activation of Th17 cells by IL-25, resulting in an increased level of IL-17 A expression, which in turn, induced the local inflammation [44]. A similar association has also been found to exist in gastrointestinal disorders. *Il25*^*−/−*^mice exhibit a higher degree of inflammation in the gastrointestinal tract, which induces elevated expression of IL-17 A [55–58]. Nevertheless, IL-25 was found to antagonize the pro-inflammatory effects of IL-17 A in both mouse models of collagen-induced arthritis (CIA), a mouse model for human RA, and experimental autoimmune encephalomyelitis (EAE), a mouse model for human multiple sclerosis (MS), thereby inhibiting the Th17 response and decreasing the degree of inflammation [9, 59, 60] (Table 1).

In conclusion, in some diseases where IL-17 A exerts pro-inflammatory effects, IL-25 may exhibit synergistic or inhibitory effects. Although IL-17RA is a co-receptor for IL-25 and IL-17 A, IL-25 requires binding to the receptor IL-17RA/IL-17RB complex, whereas the receptor complex for IL-17 A also includes IL-17RC [26]. Therefore, there may be differences between the expression of IL-25 and IL-17 A together with the associated receptors in various pathological states or target cells. However, the relationship between IL-25 and IL-17 A has not been set in stone.

#### IL-17B

IL-17B is a non-covalent dimeric glycoprotein consisting of 180 amino acids and a molecular weight of 41 kDa. IL-17B has been found to be expressed in neuronal cells, chondrocytes, germinal center (GC) B cells, and both naive and memory B cells [61]. Previous researches showed that IL-17B might share the receptor IL-17RB with IL-25 [2, 62, 63]. When researching the effect of IL-25, IL-17B is an essential cytokine.

Both IL-17B and IL-25 are expressed by colonic epithelial cells and upregulated following acute colonic inflammation. However, the deficiency in IL-25 was protective against dextran sulfate sodium (DSS)-induced colitis and a defective expression of IL-17B exacerbated the development of acute colitis. IL-25 stimulated colorectal cells to produce IL-6, an important colonic inflammatory factor, while IL-17B inhibited IL-6 expression. Furthermore, the IL-17B deficiency was found to be pathogenic for *Citrobacter rodentium* infection, which resembled DSS-induced colitis, whereas *Il25*^*−/−*^ mice were protected against *Citrobacter rodentium* infection. It was also shown that IL-25 could strongly promote allergic airway inflammation by enhancing the Th2 cell response, while IL-17B inhibited the Th2 cell response in the airways [6, 64]. IL-25 and IL-17B also perform differently in cancer. For example, IL-25 was found to inhibit the growth of MDA-MB468 breast tumor xenografts, while IL-17B promoted their growth [65, 66]. These cases show that, although IL-25 and IL-17B are both from the IL-17 family, they differ significantly in their biological function. Further, the competitive binding of IL-25 and IL-17B may lead to antagonistic effects, thereby causing different disease outcome. Therefore, when researching on the effect of IL-25, IL-17B should not be neglected.

#### IL-9

Interleukin-9 (IL-9) is a type of pleiotropic cytokine produced by a variety of cells, including Th9, Th2, Th17, and mast cells [67]. In addition, IL-9 has recently been found to contribute to the initiation and/or amplification of type 2 immune response at mucosal sites (e.g., asthma and parasitic infections) [67–70].

Current studies have shown that IL-25-stimulated dendritic cells rapidly induced mediators, such as the chemokine CCL17, which, in turn, attracted IL-9-producing T cells during allergic lung inflammation [71]. In another study, after a repeated exogenous lung infusion with IL-25, there was a large accumulation of ILC2 cells and a high production of IL-9, which further aggravated allergen-induced lung inflammation in mice [72–74]. Previous studies have demonstrated that IL-9 represents a key cytokine involved in mediating the effective expulsion of *T. spiralis* [68]. Following *Trichinella spiralis* infection, mice treated with IL-25-neutralizing antibodies failed to effectively expel *T. spiralis*. Moreover, the intensity of the antigen-specific Th9 immune response in mice was diminished, whereas the expression of IL-9 and related regulatory genes were also decreased [75]. The findings in the above study demonstrated that IL-25 could prevent the destruction by *Trichinella spiralis* through the induction of an IL-9-mediated immune response. Therefore, IL-25 can affect airway diseases and *Trichinella spiralis* infection through IL-9.

## The roles and underlying mechanisms of IL-25 in disease pathogenesis

To gain a more comprehensive understanding of IL-25, the current status of research on IL-25 in different diseases have been summarized. In particular, the following chapters focus on the mode of action and mechanisms of IL-25 in various cancers, cascade inflammation triggered by type 2 immune response, and autoimmune diseases (Table 2).

**Table 2:** The diverse roles of IL-25 in various diseases

Disease type		Role of IL-25	Ref.
**Cancer**	Hepatocellular carcinoma	Tumor supportive	[77, 85]
	Colorectal cancer		[81]
	Lung cancer		[86]
	Cutaneous T-cell lymphoma		[90]
	Melanoma	Tumor suppressive	[93]
**Autoim-mune diseases**	DSS-induced colitis in mouse	Exacerbate the severity of DSS-induced colitis.	[64, 120, 121]
	Inflammatory Bowel Disease	Express decreased in the intestine.	[55, 122]
	Rheumatoid Arthritis	Inhibit the pro-inflammatory effects and osteoclastogenesis.	[11, 124]
	Collagen-induced arthritis	Inhibit activation & differentiation from CD4^+^ T cells to Th17 cells.	[124, 125]
	Systemic Lupus Erythematosus	Positively correlated with disease activity, anti-dsDNA, and IgG.	[127, 128]
	Lupus-prone MRL/lpr	Relieve SLE symptoms.	[127]
	Type 1 diabetes mellitus	Express high in PBMC.	[130]
	Non-Obese Diabetic	Delay the recurrent autoimmune response.	[131]
	Primary Sjogren’s syndrome	Express high in peripheral blood and SG.	[131–133]
	Experimental Sjogren’s syndrome	Increase salivary flow rate.	[134]
	Multiple sclerosis	Maintain the integrity of BBB.	[136]
	Experimental autoimmune encephalomyelitis	Inhibit inflammatory factors.	[59]
**Skin diseases**	Atopic dermatitis	Promote the development and progression.	[95–[96, 111]–112]
	Psoriasis	Increases the severity.	[26, 33, 97]
	Contact dermatitis	Promote the production of associated inflammatory factors.	[44]
**Airway diseases**	Allergic airway diseases	Promote the development of airway-related diseases.	[98–103]
	Idiopathic pulmonary fibrosis	Induce fibroblast differentiation. Mediate Th2 response.	[116]
**Hepatitis**		Protect against and reverse liver injury.	[104–107]
**Obesity**		Prevent weight gain and the accumulation of lipids.	[108, 109]

### IL-25 in cancer

Although the mechanisms of cancer pathogenesis are highly complex and the causes are difficult to predict, extensive research has shown that IL-25 appears to be a critical biomarker for tumor progression [9, 76]. In addition, IL-25 can interact with multiple immune cells in a complex tumor microenvironment by initiating both innate and adaptive immune responses. The specific function of IL-25 is also dependent on different tissues or organ-specific tumors or various stages of the disease, demonstrating both tumor-supportive and tumor-suppressive effects [9].

IL-25 is distinctly dysregulated in cancer. IL-25 was detected in hepatocellular carcinoma (HCC), colorectal cancer (CRC), gastric cancer, oral squamous epithelial cell carcinoma, and multiple myeloma at a higher level compared to that of healthy individuals [77–81]. In the above cancer patients, the more advanced the cancer, the higher the level of IL-25, suggesting that IL-25 might be associated with disease progression. In contrast, IL-25 was downregulated in malignant breast and prostate cancers, it was negatively correlated with cancer severity [82, 83]. IL-25 was also low expression in the neutrophil and lymphocyte of B cell leukemia (B-CLL), and the variation in IL-25 might be associated with the development of B-CLL [84].The above results indicate that the expression of IL-25 varies substantially in different types of cancer. The dysregulation of IL-25 is also correlated with the degree of tumor cell infiltration and the prognosis of cancer patients, providing a reference for further clinical studies.

#### Mechanism of IL-25 in tumor progression

In the majority of cancers, IL-25 functions to support the tumor. During tumor progression, IL-25 primarily promotes the proliferation and metastasis of tumor cells by activating various signaling pathways, including NF-κB and STAT3 [85]. IL-25 can also contribute to the malignant proliferation and differentiation of tumor cells by interfering with cell cycle and mediating interactions in various cells [85–87].

**Fig. 3:**
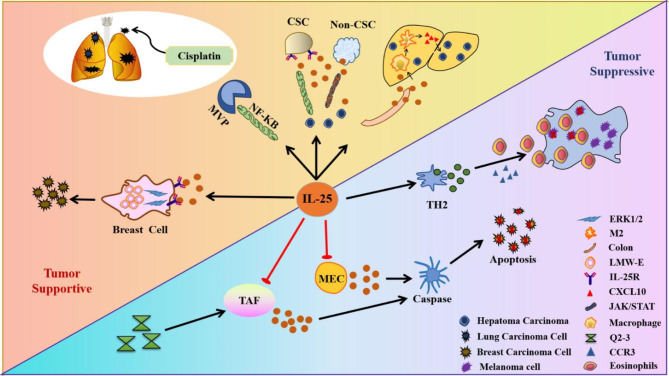
IL-25 regulates tumor progression via diverse pathways. In tumor supportive (The left), IL-25 could induce MVP to aggravate chemoresistance. IL-25 might promote tumor proliferation via LMW-E. And also, IL-25 promotes cancer stem cell self-renewal by inducing JAK/STAT3 signaling pathway. IL-25 could induce macrophages to secrete the chemokine CXCL10, which in turn activates the EMT. In tumor suppressive (The right), IL-25 mainly induces apoptosis in tumor cells by activating caspase enzymes. IL-25 contributes to promote the entry of infiltrating eosinophils into the tumor microenvironment and mediates the killing of tumor cells. MEC, refer in particular to nonmalignant mammary epithelial cells. Black arrow indicates that IL-25 promotes the reaction, red arrow indicates that IL-25 inhibits the reaction. The image is produced by Adobe Illustrator

In HCC, dysbiosis of gut microbiota results in hyperplasia of tuft cells, these cells would secret large amounts of IL-25. Then IL-25 might stimulate macrophages to release CXCL10, which then activated the epithelial-mesenchymal transition (EMT) pathway [77]. Through the study of HCC cell lines (Huh7, PLC/PRF/5, HCC cells T1115 and T1224) and models, researchers found non-cancer cell stem cells (non-CSCs) could secrete IL-25 into the tumor microenvironment, and secreted IL-25 interacts with IL-17RB on CSCs. [85] (Fig. 3). Resistance to chemotherapy in lung cancer patients might also be affected by IL-25. Exogenous IL-25 can stimulate the expression of Major vault protein (MVP) by activating the NF-κB signaling pathway. Since MVP causes chemoresistance in lung cancer, an increase in IL-25 and MVP would greatly aggravate chemotherapy resistance in lung cancer patients [86, 88, 89] (Fig. 3). Therefore, interference with IL-25 might represent a potential therapeutic strategy for the clinical reversal of chemotherapy resistance. In cutaneous T-cell lymphoma (CTCL), Th2 cytokines and periostin induced IL-25 produced by epidermal keratinocytes. Secreting IL-25 into the epidermis activated IL-25R-expressing T cells. Then IL-25 bound to IL-25R to promote the polarization of Th2 cells by activating the phosphorylated STAT6 pathway. Subsequently, Th2 cells secreted large amounts of IL-4, IL-5, and IL-13 to augmented and created a TH2-dominant microenvironment. The microenvironment would inhibit antitumor TH1 responses and promote CTCL [90]. IL-25 might promote tumor progression by activating multiple signaling pathways and causing downstream cell signaling cascades.

In CRC patients, the levels of IL-25 in serum and tissue were significantly increased. In the CRC mouse model, the IL-25-ILC2 axis could activate type 2 immune response to shape the tumor microenvironment.The IL-25-ILC2 axis also promoted the immunosuppressive effect of myeloid-derived suppressor cells (MDSCs) on CD8^+^ T cells in the colon. This led to a depletion of tumor-infiltrating CD8^+^ T cells and exacerbate colorectal cancer [81]. In summary, the IL-25-ILC2 pathway may serve as a novel therapeutic target against CRC in the future. IL-25 may also promote the malignant proliferation of breast cancer cells. Study found that c-RAF, ERK and p70S6 kinases were phosphorylated in breast cancer cells following treatment with IL-25 in several breast cancer cell lines (T47D, MCF7, BT-20, and IJG-1731). Activation of the above pathways can promote the accumulation of the low molecular form of cyclin E (LMW-E). Increased LMW-E has been demonstrated to accelerate the G1/S transition, promote malignant proliferation, and differentiate breast cancer cells [85, 87, 91] (Fig. 3). Thus, IL-25 is capable of promoting tumor development by activating multiple mechanistic pathways and affect different target cells in the complex tumor microenvironment. These findings could also provide a reference for the clinical treatment of related tumors.

#### Mechanisms of IL-25 in tumor suppression

Although current studies indicate that IL-25 is primarily a tumor-promoting factor, IL-25 may hinder tumor progression under certain conditions. The study suggested IL-25 might be secreted from tumor-associated fibroblasts (TAFs). Synthetic dihydrobenzofuran lignan (Q2-3) could induce elevated IL-25 secretion and increase fibroblastic IL-25 activity. The above approaches were effective in suppression of mouse 4T1 mammary tumor metastasis (a mouse model of breast cancer). [92]. Nonmalignant mammary epithelial cells (MECs) in conditioned medium could produce large amounts of IL-25 following differentiation. Subsequently, IL-25 inhibited the proliferation of breast cancer cells. IL-25 could also initiate death signals in breast cancer cells by recruiting TRADD and FADD to induce caspase-mediated apoptosis [66] (Fig. 3). In the melanoma model, IL-25 induced the production of IL-5 by Th2 cells. In the tumor microenvironment, IL-5 activated CC motif chemokine receptor type 3 (CCR3). CCR3 can induce the chemotaxis of eosinophils in the blood and spleen of tumor-bearing mice to infiltrate into the tumor site and exert anti-tumor activity [93] (Fig. 3).

In conclusion, large amounts of studies indicate a role of IL-25 in tumor immunity. Since most studies on the mechanism of IL-25 in tumor development have been conducted in animal models and cell lines, the precise effect of IL-25 in human cancers requires further investigations. Therefore, translational studies are required to determine the biological function of IL-25 in cancer development.

### IL-25 in allergic inflammation and inflammatory diseases

Accumulating evidence suggests that IL-25 is extensively expressed in various epithelial cells. As a “barrier surface” cytokine, the production of IL-25 is dependent on external stimuli or microenvironmental factors [53, 54]. The upregulation of IL-25 may lead to inflammatory diseases, including atopic dermatitis, psoriasis or asthma. IL-25 is involved in enhancing the severity of allergic reactions through the induction of a type 2 immune response [94–103]. IL-25 also plays a role in inflammatory diseases including obesity and digestive system disorders [104–109].

#### IL-25 in skin diseases

Atopic dermatitis (AD) is a chronic, relapsing inflammatory skin disease that is often associated with polyfilamentous protein mutations [110]. After stimulated by house dust mites, the expression of IL-25 in human keratinocytes was increased [111]. The production of IL-25 from dermal dendritic cells in AD patients might inhibit the synthesis of polysilk protein, which directly disrupts the function of the skin barrier [112]. IL-25 also increased the expression of pruritogenic endothelin 1 in keratinogenic cells via the ERK1/2 and JNK pathways, which contributed to the degree of pruritus during the onset of AD [94]. In the epidermis of mouse models and patients,when suffered from acute and chronic allergic skin inflammation, stratum corneum cells also produced large amounts of IL-25 in the inflammatory environment [95, 96]. IL-25 bound to ILC2 cells increased the infiltration of CD4^+^ T cells and promoted the development of inflammation [95]. Therefore, IL-25 is an important inflammatory factor that has been found to promote the development and progression of AD.

Psoriasis is a chronic inflammatory skin disease [113]. Psoriasis has been found to be associated with higher level of IL-25 in patients compared to normal subjects [32]. Moreover, the researchers genotyped single nucleotide polymorphisms (SNPs) in psoriasis patients and healthy controls, the *IL17E* rs79877597 SNP was a modifier of the risk for psoriasis disease severity and psoriatic arthritis [97]. An intradermal injection of recombinant IL-25 into the ear or dorsal skin of mice was identified to induce psoriasis-like pathology, including epidermal acanthosis and dermal thickening, dermal immune cell infiltration, and pustule formation in the mouse model [26]. The above findings emphasize that IL-25 represents a prominent factor in the development of skin inflammation in psoriasis and is expected to be a potential target for clinical treatment in the future.

Additionally, patients with contact dermatitis have also been observed to exhibit a significant increase in IL-25 expression, which could promote the production of associated inflammatory factors (e.g., IL-5 and IL-13), which in turn led to local inflammation [44]. The above studies demonstrate that IL-25 is one of the critical factors in skin inflammation.

#### IL-25 in airway diseases

Allergic airway diseases (AADs) constitute a heterogeneous group of diseases mediated by the Th2 immune response, including allergic asthma, allergic rhinitis, and chronic sinusitis. Bronchus and lung epithelia upregulated the production of IL-25 in response to various stimuli including allergens, fungal antigens, and Toll-like receptor ligands [98, 99]. In asthma patients, IL-25 has been found to increase the expression of endothelial VEGF/VEGF receptors through the PI3K/AKT and ERK/MAPK pathways. Activation of these pathways can promote angiogenesis and exacerbate the development of asthma [98]. Instead, blocking IL-25 significantly reduced the antigen-induced infiltration of eosinophils and CD4^+^ T cells in the airway [100]. In a mouse model of asthma, IL-25 was found to stimulate natural killer T cells to produce inflammatory factors (e.g., IL-13), thereby promoting airway hyperresponsiveness [101]. The combined activity of IL-25 and IL-33 could further enhance the Th2 response in patients with chronic rhinitis and nasal polyposis [102]. After a combined blockade of IL-25, IL-33, and TSLP, the expression of inflammatory factors and level of airway fibrosis were reduced in the mouse model [103]. In allergic rhinitis, as a kind of nasal epithelial-derived proinflammatory cytokines, IL-25 mainly promoted the production of type 2 cytokines IL-5 and IL-13 from Th2 and ILC2, but the pathway may be affected by IL-35. IL-35 can inhibit the production of IL-25 from human nasal epithelial cells induced by *Dermatophagoides pteronyssinus* and *Aspergillus fumigatus* [114, 115]. In conclusion, IL-25 could regulate the occurrence of type 2 immune responses in a variety of innate or adaptive cells and promote the development of airway-related diseases.

On the other hand, IL-25 plays a critical role in epithelial-mesenchymal crosstalk and local tissue remodeling. When these responses are dysregulated, it can cause tissue fibrosis and lead to many lung diseases. IL-25 was upregulated in the alveolar epithelial cells and lung fibroblasts of patients with idiopathic pulmonary fibrosis. The upregulation of IL-25 was positively correlated with the degree of inflammatory infiltration and fibrosis [116]. IL-25 can directly induce fibroblast differentiation to disrupt the lung environment and mediate Th2 response to produce large quantities of pro-inflammatory cytokines (e.g., IL-5 and IL-13). Subsequently, inflammatory factors induced fibroblasts to further accumulate and differentiate, resulting in exacerbated pulmonary fibrosis [117, 118]. Therefore, IL-25 may be a potential target for the treatment of airway and lung-related diseases.

#### IL-25 in other inflammatory diseases

In hepatitis patients and a mouse model of fulminant hepatitis, IL-25 was found to protect against and reverse liver injury by promoting an increase in MDSCs, which effectively inhibited the activation of immune cells [104, 105]. In addition, following intestinal helminth infection, IL-25 regulated type 2 cytokines to alleviate chronic inflammation in the gastrointestinal tract [106, 107]. Therefore, IL-25 plays a role in combating inflammation in the digestive system.

The administration of IL-25 to high-fat diet (HFD)-fed wild-type mice significantly improved hepatic steatosis [108]. In the obese mouse models, IL-25 enhanced lipid uptake by macrophages and also increased the mitochondrial respiratory capacity and oxygen consumption rate of macrophages. Thus, IL-25 can effectively prevent weight gain and the accumulation of lipids [109]. The above studies demonstrate the potential of IL-25 for the treatment of obesity and related metabolic syndrome.

### IL-25 in autoimmune diseases

During the development of autoimmune diseases (e.g., systemic lupus erythematosus [SLE], RA, inflammatory bowel disease [IBD], and Sjogren’s Syndrome [SS]), autoimmune responses can lead to inflammation and tissue damage [2]. IL-25 is actively involved in the progression and prognosis of autoimmune diseases.

#### Inflammatory bowel disease

**Fig. 4:**
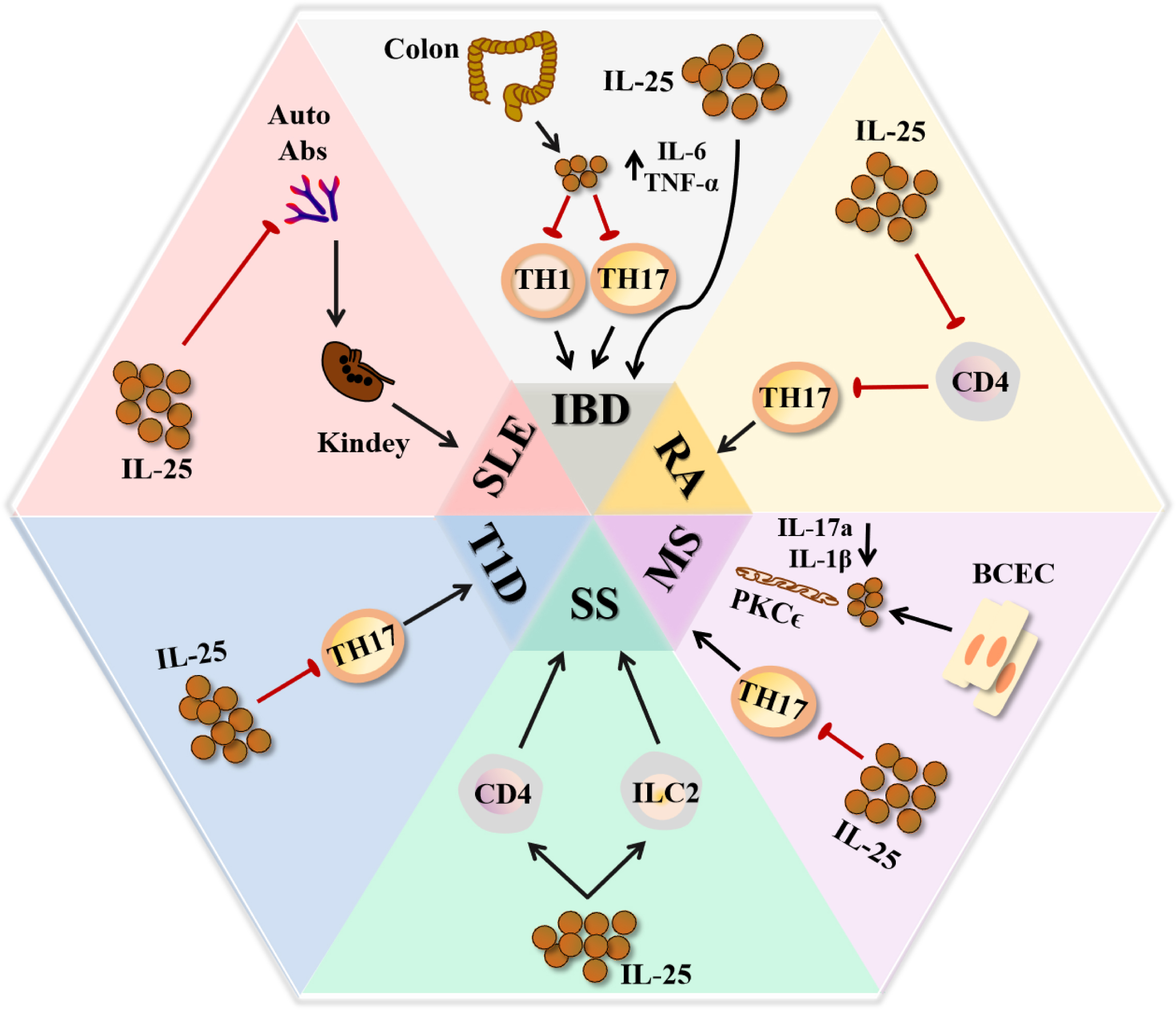
The roles of IL-25 in autoimmune diseases. IL-25 can alleviate the progression of SLE by inhibiting the production of autoantibodies. In RA, T1D, and MS, IL-25 might exert anti-inflammatory effects by inhibiting the Th17 response. In MS, IL-25 could downregulate the expression of IL-1β and IL-17 A through the phosphorylation of PKCϵ phosphorylation pathway. IL-25 is involved in the pathogenesis of IBD, but IL-25 can also inhibit the cellular activity of Th17 and Th1 in IBD patients. In SS, IL-25 could exacerbate the disease severity. Black arrow indicates the reaction occurs as usual, red arrow indicates the reaction is obstructed. The image is produced by Adobe Illustrator

IBD is a type of chronic inflammatory disorder affecting the gastrointestinal tract. IBD patients may present with symptoms, such as chronic diarrhea, rectal bleeding, abdominal pain, and weight loss [119]. It was found that *Il25*^*−/−*^mice treated with DSS were associated with significantly lower weight loss, colonic ulceration, and histological score compared to the control group. Exogenous IL-25 could upregulate the expression of relevant inflammatory factors, such as IL-33, IL-6 and TNF-α, in colonic epithelial cells. This experiment suggested that IL-25 could exacerbate the severity of DSS-induced colitis [64, 120, 121]. While IL-25 could promote the development of IBD in animal models, the findings in patients with IBD are contrary. Studies found that IL-25 was decreased in the intestine from some IBD patients compared to healthy individuals, which may affect the expansion of Th17 and Th1 cells in the intestine [55, 122]. Therefore, further clinical studies are pending to verify the precise effect of IL-25 in patients with IBD (Fig. 4).

#### Rheumatoid arthritis

RA is a chronic systemic autoimmune disease, which leads to joint deformity and loss of function [123]. Clinical studies have found that the level of IL-25 was upregulated in the serum and synovial fluid of RA patients [124]; however, the increase of IL-25 contributed to the reduction of inflammatory response and disease severity in RA patients. In studies of RA patients, IL-25 was found to inhibit the pro-inflammatory effects of IL-17 A by competing for binding to the IL-17RA receptor, but could also inhibit osteoclastogenesis through STAT3 and p38MAPK pathways in RA [11, 59]. In mice, IL-25 could inhibit the activation and differentiation from CD4^+^ T cells to Th17 cells, which in turn attenuated the progression of CIA (Fig. 4). However, the level of IL-25 was found to be significantly higher in CIA mice compared to that in wild-type mice as the disease progresses [124, 125]. Therefore, the exact role of IL-25 in RA requires more researches.

#### Systemic lupus erythematosus

SLE is an autoimmune inflammatory disease involving multiple organs, with lupus nephritis as the most serious complication [126]. Previous studies have shown that IL-17 A was critically involved in the progression of SLE [127]. Several studies reported that the serum level of IL-25 was higher in patients with SLE, especially in patients with lupus nephritis. In particular, IL-25 was positively correlated with disease activity, anti-dsDNA, and IgG [127, 128]. Although IL-25 may be involved in disease progression in SLE patients, high level of IL-25 in lupus mice acts a protective effect on disease progression. Administration of IL-25 relieved SLE symptoms in lupus-prone MRL/lpr mice, including the decline of anti-dsDNA and IgG, and the degree of kidney damage was also reduced. Additionally, the expression of relevant inflammatory factors (e.g., IL-1α, IL-1β, and IFN-β) was decreased in lupus-prone MRL/lpr mice [127] (Fig. 4). Thus, the precise role of IL-25 in the development of SLE still requires future investigation.

#### IL-25 in other autoimmune diseases

Type 1 diabetes mellitus (T1D) is a metabolic disorder syndrome [129]. In a study of T1D patients, the expression of IL-25 was found to be noticeably increased in the peripheral blood mononuclear cells (PBMC) of T1D patients compared to healthy individuals [130]; however, exogenous IL-25 could restore blood glucose levels in newly diabetic animals, and notably delay the recurrent autoimmune response after islet transplantation in the Non-Obese Diabetic (NOD) mouse model. Some studies have also found that IL-25 might alleviate T1D by inhibiting the Th17 response [56, 131] (Fig. 4). Therefore, further clinical studies are required to investigate the effect of IL-25 on the pathogenesis of T1D.

Primary Sjogren’s syndrome (pSS) is a chronic autoimmune disease characterized by dry eyes, dry mouth, and other clinical manifestations [132, 133]. The level of IL-25 in peripheral blood and salivary gland (SG) was particularly high in patients with pSS. Following IL-25 stimulation, PBMC from pSS patients displayed a higher level of autoantibodies. Moreover, IL-25 was detected in tissue cells in the submandibular gland, mostly acinar and ductal cells in mice. The quantity of IL-17RB^+^ ILC2 cells in SG and peripheral blood were also increased in vitro and in a mouse model. The salivary flow rate of experimental Sjogren’s syndrome mice was enhanced following anti-IL-25 therapy. These findings suggest that IL-25 might be a potential therapeutic target for SS [134] (Fig. 4).

Multiple sclerosis (MS) is the most common type of central nervous demyelinating disease, occurring in the optic nerve, spinal cord, and brainstem [135]. *Il25*^*−/−*^ mice were highly susceptible to EAE. After the knockdown of IL-25, the numbers of inflammatory IL-17 A, IFN-γ, and TNF-α-producing T cells that invaded the central nervous system (CNS) were increased in EAE mice. Th17 and Th1 cell-mediated inflammatory responses were also suppressed, the degree of inflammation in EAE mice was alleviated following exogenous IL-25 treatment [59]. Study found that IL-25 produced from brain capillary epithelial cells (BCEC) during the pathogenesis of MS could help maintain the integrity of the blood-brain barrier (BBB). IL-25 might downregulate the expression of related inflammatory factors (IL-1β and IL-17 A) through the phosphorylation of protein kinase C epsilon (PKCϵ) phosphorylation pathway [136] (Fig. 4). Therefore, IL-25 may serve as a mitigating agent in MS.

## Therapeutic targeting for clinical treatment

Recent studies have indicated that selective blockade of IL-25 may represent a promising therapeutic approach for treating inflammatory diseases and cancers.

In a highly malignant spontaneous breast tumor model, a blockade of IL-25 suppressed tumor-infiltrating CD4^+^ T cells and macrophages, to prevent breast tumor invasion and subsequent lung metastasis [137]. Cisplatin is a powerful anticancer reagent frequently used to treat different types of solid tumors. Cisplatin down-regulates IL-25 and IL-25R in patients, which effectively blocked the promotional effects of IL-25 on cervical cancer cell viability, migration, and invasion [138]. In addition, the administration of anti-IL-25 monoclonal antibodies during the sensitization phase in mice with allergic asthma was effective in relieving symptoms, including decreased levels of IgE in the serum, suppression of eosinophil infiltration, and a significant decrease in airway hyperresponsiveness [139, 140]. Combined blockade treatment with soluble IL-25R and IL-13R protein inhibitors significantly suppressed innate and adaptive immune responses and promoted tissue remodeling in asthmatic mice [140]. Similar findings have also been found in autoimmune diseases. In studies related to psoriasis treatment, bromotalizumab effectively resisted IL-17RA, which would prevent the signaling of IL-25 and IL-17 A [141–144]. Rituximab was conformed to treat pSS by blocking the expression of IL-25 [92]. In conclusion, these findings indicate that selective blockage of IL-25 is an effective treatment for a variety of diseases, which may provide supportive evidence for clinical trials in the future.

Currently, most studies on the selective blockade of IL-25 have focused on animal models and preclinical investigations, further clinical investigations on the roles of IL-25 in various inflammatory disorders and cancers are needed to validate IL-25 as a therapeutic target in the future.

## Conclusion

As a member of the IL-17 family, IL-25 binds to the receptor complex (IL-17RA/IL-17RB) and exerts different effects in various types of diseases. Moreover, IL-25 can activate a series of signaling pathways, including the NF-κB, MAPK, and JAK pathways. IL-25 from various cells (e.g., Th2 cells and various epithelial cells) can activate many immune cells and induce the release of pro-inflammatory cytokines, which further contributes to the progression of tumors and various inflammatory diseases. In addition, the dysregulation of IL-25 signaling participates in cancer progression by affecting cell cycle changes, resisting apoptosis, and assisting pro-tumor cytokines. IL-25 can also promote the infiltration of eosinophils and CD4^+^ T cells into the airways and skin by promoting a type 2 cytokine response, which may even induce pulmonary fibrosis during allergic inflammation. In autoimmune diseases, IL-25 is elevated and associated with an increased level of autoantibodies in the peripheral blood of patients. It has been shown that IL-25 exacerbates autoimmune disease progression in many mice models, including SS and IBD.

The positive effect of IL-25 in numerous allergy and respiratory disorders has currently been established. Since most studies on the mechanism of IL-25 in tumor development have been conducted in animal models and cell lines, the exact role of IL-25 in human cancer still needs to be supported by a large amount of clinical data. Numerous earlier studies have shown the immunomodulatory potential of IL-25 in disease pathogenesis, and IL-25 is expected to be a biomarker that reflects the severity or progression of different diseases. IL-25 is involved in a variety of diseases (cancer, inflammation, and autoimmune diseases), so IL-25 has potential in explaining disease pathways, drug/disease interactions, and offering reference for associated research of clinical treatment. However, clinical application of targeting IL-25 is still lacking. Blocking IL-25 or its receptors may be considered in future clinical trials. The use of combination therapies based on IL-25 may show promise in related diseases. Currently, a phase I clinical trial is undergoing to evaluate the safety of blocking IL-25 in healthy adults (NCT05128409). Further study is needed to validate the efficacy of IL-25-targeted therapies for treating various diseases.

## Data Availability

Not applicable.

## Abbreviations

IL-25: Interleukin-25
Th2: T helper 2
TSLP: Thymic stromal lymphopoietin
IL-17R: IL-17 receptor
IL-25R: IL-25 receptor
IL-17Rh1: IL-17 receptor homolog 1
KO: Knockout
SEF: Similar expression to fibroblast growth factor genes
SEFIR: SEF/ IL-17 receptor
NF-κB: Nuclear factor-κB
Act1: NF-κB Activator 1
TRAF: Tumor necrosis factor receptor-associated factor
AP-1: Activator protein 1
BATF: Basic leucine zipper transcription factor, activating transcription factor-like
ILC2: Group 2 innate lymphoid cell
DAZAP2: Deleted in Azoospermia-associated protein 2
SMURF2: Smadubiquitin regulatory factor 2
TNFR1: Tumor necrosis factor receptor 1
TRADD: TNFR1 associated death domain protein
FADD: Fas-associated death domain
CysLT: Cysteinyl leukotriene
NFAT: Nuclear factor of activated T cell
RA: Rheumatoid arthritis
CIA: Collagen-induced arthritis
EAE: Experimental autoimmune encephalomyelitis
GC: Germinal center
DSS: Dextran sulfate sodium
IL-9: Interleukin-9
HCC: Hepatocellular carcinoma
CRC: Colorectal cancer
EMT: Epithelial-mesenchymal transition
CSC: Cancer cell stem cell
MVP: Major vault protein
CTCL: Cutaneous T-cell lymphoma
MDSC: Myeloid-derived suppressor cell
LMW-E: Low molecular form of cyclin E
Q2-3: Synthetic dihydrobenzofuran lignan
MEC: Mammary epithelial cell
CCR3: CC motif chemokine receptor type 3
TAFs: Tumor-associated fibroblasts
AD: Atopic dermatitis
AAD: Allergic airway disease
HFD: High-fat diet
SLE: Systemic lupus erythematosus
IBD: Inflammatory bowel disease. SS:Sjogren’s Syndrome
T1D: Type 1 diabetes mellitus
PBMC: Peripheral blood mononuclear cell
NOD: Non-Obese Diabetic
pSS: Primary Sjogren’s syndrome
SG: Salivary gland
MS: Multiple sclerosis
CNS: Central nervous system
BCEC: Brain capillary epithelial cell
BBB: Blood-brain barrier
PKCϵ: Protein kinase C epsilon
SNP: Single nucleotide polymorphism
